# Clinical Features and Serological Markers Risk Model Predicts Overall Survival in Patients Undergoing Breast Cancer and Bone Metastasis Surgeries

**DOI:** 10.3389/fonc.2021.693689

**Published:** 2021-09-17

**Authors:** Haochen Mou, Zhan Wang, Wenkan Zhang, Guoqi Li, Hao Zhou, Eloy Yinwang, Fangqian Wang, Hangxiang Sun, Yucheng Xue, Zenan Wang, Tao Chen, Xupeng Chai, Hao Qu, Peng Lin, Wangsiyuan Teng, Binghao Li, Zhaoming Ye

**Affiliations:** ^1^Department of Orthopedic Surgery, The Second Affiliated Hospital, Zhejiang University School of Medicine, Hangzhou, China; ^2^Orthopedics Research Institute of Zhejiang University, Hangzhou, China; ^3^Key Laboratory of Motor System Disease Research and Precision Therapy of Zhejiang Province, Hangzhou, China

**Keywords:** breast cancer, bone metastasis, prognosis, surgeries, Cox regression, nomogram

## Abstract

**Background:**

Surgical therapy of breast cancer and bone metastasis can effectively improve the prognosis of breast cancer. However, after the first operation, the relationship between preoperative indicators and outcomes in patients who underwent metastatic bone surgery remained to be studied. Purpose 1. Recognize clinical and laboratory prognosis factors available to clinical doctors before the operation for bone metastatic breast cancer patients. 2. Develop a risk prediction model for 3-year postoperative survival in patients with breast cancer bone metastasis.

**Methods:**

From 2014 to 2020, patients who suffered from breast cancer bone metastasis and received therapeutic procedures in our institution were included for analyses (n=145). For patients who underwent both breast cancer radical surgery and bone metastasis surgery, comprehensive datasets of the parameters of interest (clinical features, laboratory factors, and patient prognoses) were collected (n=69). We performed Multivariate Cox regression to identify factors that were associated with postoperative outcome. 3-year survival prediction model and nomograms were established by 100 bootstrapping. Its benefit was evaluated by calibration plot, C-index, and decision curve analysis. The Surveillance, Epidemiology, and End Results database was also used for external validation.

**Results:**

Radiotherapy for primary cancer, pathological type of metastatic breast cancer, lymph node metastasis, elevated serum alkaline phosphatase, lactate dehydrogenase were associated with postoperative prognosis. Pathological types of metastatic breast cancer, multiple bone metastasis, organ metastases, and elevated serum lactate dehydrogenase were associated with 3-year survival. Then those significant variables and serum alkaline phosphatase counts were integrated to construct nomograms for 3-year survival. The C-statistic of the established predictive model was 0.83. The calibration plot presents a graphical representation of calibration. In the decision curve analysis, the benefits are higher than those of the extreme curve. The receiver operating characteristic of the external validation of the model was 0.82, indicating a favored fitting degree of the two models.

**Conclusion:**

Our study suggests that several clinical features and serological markers can predict the overall survival among the patients who are about to receive bone metastasis surgery after breast cancer surgery. The model can guide the preoperative evaluation and clinical decision-making for patients. Level of evidence Level III, prognostic study.

## Introduction

Breast cancer (BC) is the most common malignancy among women (1, 2), and bone is the most common location of metastasis in BC patients. There are about three-quarters of stage IV BC patients developing skeletal metastases (3, 4). Poor survival, skeletal-related events (SREs) (regarded as the demand for surgery and radiotherapy to the bone, intractable pain, pathological fracture, hypercalcemia, and spinal cord compression), reduced quality of life, and considerable morbidity are the consequences of BC bone metastasis (5).

The median overall survival (OS) of BC patients from bone metastasis diagnosis is 40 months (5). The 3-year survival rate of BC patients with bone metastasis was 25%, and the 5-year was 13% (6). While premature death is inevitable, remission is often performed with surgery, chemotherapy, and the development of hormone or bone-targeted drug therapies. The life quality of BC patients, including bone metastasis BC patients, improved (2).

The primary site decides the prognosis of metastatic bone disease, with breast and prostate cancers associated with survival measured in years compared with lung cancer. The average survival is only a few months (7). Poorer mean survival was found in the bone metastasis patients with non-small cell lung cancer (NSCLC) (8). Besides, the presence of extraosseous disease and the extent and tempo of the bone disease are potent predictors of prognosis. The former is usually estimated based on the features of the original tumor and common tumor markers. Simultaneously, the latter is often evaluated based on bone-specific markers and clinical manifestations of the bone. Our better comprehension of prognosis and predictors may lead to a more personalized treatment for each patient and more cost-effective healthcare resources.

Some researchers reported that local surgery might obtain advancement in the survival of metastatic BC (9, 10). Recently, Hou et al. also proposed that surgery of the primary lesion could help prolong the survival of patients and established a nomogram for bone metastasis of breast cancer (11). Nomogram is commonly used to predict the prognosis of cancer patients and has been used in survival studies of metastatic BC (11–14). Under such conditions, we are more interested in the survival of patients who have bone metastases and experience subsequent surgery. However, few similar studies have been reported.

In the current study, we aimed to recognize clinical and laboratory prognosis factors available to clinical doctors before the operation for bone metastatic breast cancer patients and construct a prognostic nomogram for BC bone metastasis.

## Methods

### Study Design and Patients

Ethics Committee of the Second Affiliated Hospital of Zhejiang University School of Medicine review board approved this retrospective study, which was conducted in accordance with the principles of the Helsinki Declaration. All medical record data were collected at our medical centres. After surgery for breast cancer, patients who had subsequent surgery for bone metastases were included.

**Figure 1:**
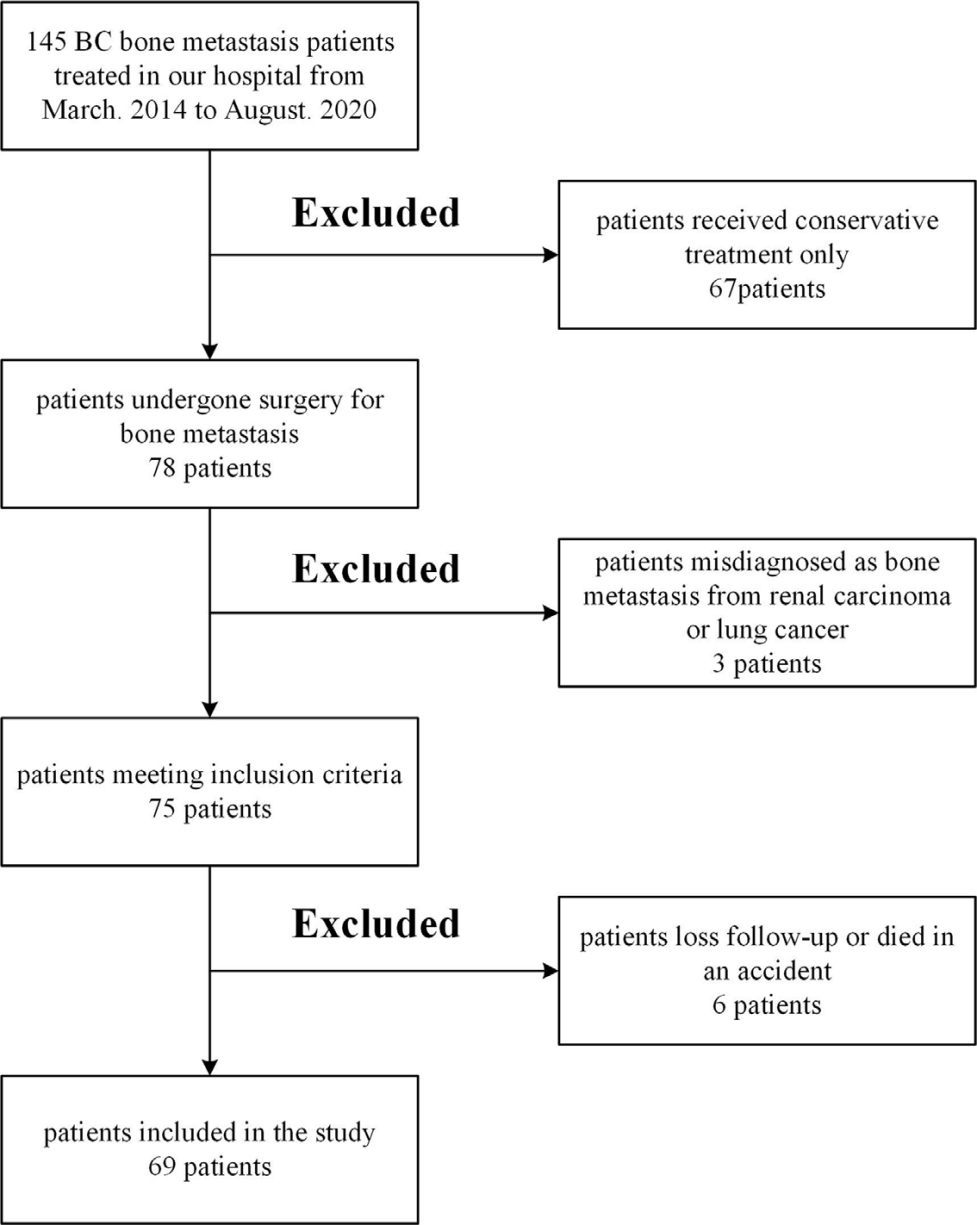
Flow chart of the study.

This was a retrospective study based on patients’ records. From March. 2014 to August. 2020 we treated 145 BC patients with bone metastasis in the institution. We excluded 67 patients who received only conservative therapy, three patients diagnosed with bone metastasis from renal carcinoma or lung cancer, one patient who died in an accident, five patients of less than six months follow-up or loss of contact. Patients with pathology that might affect the assessment of risk factors, such as liver dysfunction and other malignancies, were excluded. The remaining 69 patients met the standards, including metastatic bone events following radical breast cancer surgery and completed surgery at our institution with a minimum follow-up time of 6 months. **Figure 1** shows the detailed flow chart of the study. In these groups, comprehensive data sets for the relevant parameters (including clinical factors, laboratory factors, tumor markers, and survival information) were provided. Five experienced surgeons performed all operations.

The exclusion criteria for the study are as follows:

(1) No surgery was performed for the primary lesions and metastases.
(2) Information on radiotherapy and chemotherapy was missing.
(3) Clinical features and preoperative laboratory examination were missing.
(4) Visceral and brain metastases status was unclear.
(5) Other systemic diseases and accidental deaths.
(6) Follow-up time less than six months.

### Description of Treatment

Due to the limitations of this retrospective study, we did not define the surgical method preoperatively at that time. Tumor curettage and internal fixation or extensive segment resection and replacement were performed according to the Mirels’ scoring system for long bone (15) and the Tomita scoring system for the spine (16). Surgical indications involved a failure to improve after several conservative treatments. Regarding indications, pathological or impending limb fracture is a vital indicator of the quality of life recovery. Isolated metastasis patients in whom prolonged survival is expected is also an indication for surgery. Acute paralysis of the spinal cord due to the spinal metastases’ acute collapse would be one such implication.

**Figure 2:**
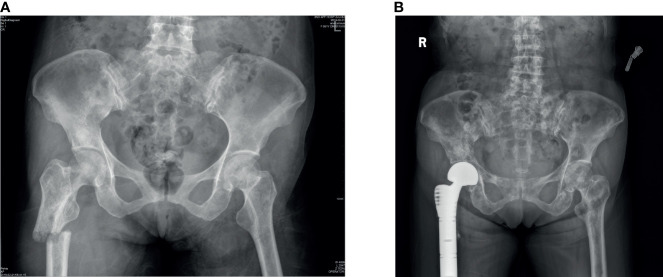
**(A)** As we can observe from the x-rays that a patient had bone metastases from breast cancer and a pathological fracture of the right femur. **(B)** The patient underwent right hip replacement surgery.

All patients underwent radical surgery for breast cancer and were confirmed to be primary breast cancer by histopathological analysis of specimens obtained from surgery. Bone metastasis can be diagnosed by a bone scan (**Figure 2**), and other organ metastases can be diagnosed by plain radiographs, CT, or MRI. We obtained the pathological specimens of these patients after surgery in our hospital and performed immunohistochemistry on them. All these examinations were completed in the auxiliary department of our institution, including the examination of blood indicators.

### Variables, Outcome Measures, Information Source, and Bias

Our primary outcome was overall patient survival after surgery. Total survival (OS) was calculated from the initial surgery date to tumor death or last follow-up. The date of death was collected from the patient’s family through follow-up.

Our secondary outcome was 3-year survival after surgery. Based on the patients’ follow-up information, patients who were followed for longer than three years and died within three years were included (n=44).

We assessed clinical characteristics obtained from the patient’s electronic medical record system. We were reconfirmed at follow-up, which included the patients’ age at diagnosis of the prime tumor and metastasis, disease-free interval (DFI), treatment for BC (chemotherapy, radiotherapy, and endocrine therapy), metastasis positions and number, surgery method (radical surgery or palliative surgery). Radiological reports were evaluated to assess the presence of bone, brain, lung, or liver metastases and the event of pathological fractures. Again, we authenticated this at follow-up. Pathological findings of bone metastases such as molecular expression of metastasis (ER status, PR status,her2 status), ER expression intensity(0-10%,10-60%,60-100%) were obtained from Pathology Department. We classified the metastases (LuminalA, LuminalB, her2-enriched, triple-negative) according to the pathological results (17). Although this could be different from the pathology of primary cancer (18), it showed the pathology of bone metastases, which might better conduct the therapy.

Blood routine examination, serum electrolytes, tumor marker, were obtained from the Laboratory Department of our hospital. All patients in this study were tested for blood markers on admission and underwent immunologic analysis of the specimens postoperatively to obtain these complete data. For patients with multiple tests before the operation, the most recent preoperative results were selected.

We chose patients from Surveillance, Epidemiology, and End Results (SEER) database (https://seer.cancer.gov/) for external verification. The same standard case (n=108) were selected as our center for external verification. The indicators for verification include organ metastasis, hormone receptor, age, radiotherapy and chemotherapy of primary foci.

### Statistical Analysis

We depicted survival curves using the Kaplan-Meier method and compared them using the log-rank test to have an initial understanding of risk factors. We applied multivariate Cox regression analysis of the patients stratified by the incidence of postoperative death. First, the possible variables were selected from the 41 variables (univariate analysis p < 0.1). According to clinical significance, variables that may have a collinear relationship were excluded, and the study’s unrelated variables were excluded too. Multivariate Cox regression analysis was performed on the selected variables, and a stepwise procedure commanded confounding variables.

As for the establishment of a risk model, first, we combined it with the Stepwise Logistic Regression method based on Akaike’s Information Criterion (19) for the sake of the best model. Secondly, we formulated a nomogram. The efficiency of the nomogram was judged by concordance index (C-index), and the discriminant performance of the model was measured using C-statistics, which varies from 0.5 (random forecast) to 1.0 (excellent distinction) (20). In the calibration plot, calibration could be envisioned. The prediction probability of the result is overestimated when the correction intercept is less than 0. While when the intercept is positive, the algorithm is underestimated (21, 22). Meanwhile, we drafted a decision curve analysis to show the net benefit of different models. The “None” line would show the expected net benefit if the interference changes were not performed. The “ALL” line presents the expected net benefit for all patients with the intervention development (20).

Data analysis, curve drawing, and model establishment were performed with Microsoft Excel (Redmond, WA, USA), the IBM SPSS Statistics 23 (La Jolla, CA, USA), R version 4.0.3 (The R Foundation, Vienna, Austria).

## Result

### Demographics, Description of Study Population

We analyzed the electronic medical record systems of 69 enrolled women patients (**Table 1**). All of them had experienced two operations (surgery for breast cancer and surgery for bone metastases). The median postoperative follow-up time was 24 months (IQR 12-31 months). The median age at the diagnosis of breast cancer was 49 years (IQR, 44-55), and the median age at the diagnosis of bone metastasis was 53 years (IQR, 49-62), the median time from the primary diagnosis to the operation of bone metastasis was 5 years (IQR, 2-8). In total, 67% (46 patients) had multiple bone metastasis, and 28% (19 patients) had other organ metastasis. Meanwhile, 46% (32 patients) had a pathologic fracture. 54% (37 patients) underwent palliative surgery, while 46% (32 patients) underwent radical surgery.

**Table 1:** Characteristics of participants.

Categorical variables	Values (%)	Continuous variables	Median (interquartile range)
Chemotherapy for breast cancer	60 (87%)	Age at primary cancer (years)	49 (44-55)
Radiotherapy for breast cancer	47 (68%)	Age at metastasis cancer (years)	53 (49-62)
Endocrine therapy for breast cancer	25 (36%)	Disease free interval (DFI) (years)	5 (2-8)
Luminal A	7 (10%)		
LuminalB	35 (50%)	CEA (U/L)	5.7 (2.5-44.4)
Her2-enhanced	8 (12%)	CA125 (U/L)	17.3 (10.4-47)
Triple-negative	19 (28%)	CA153 (U/L)	31.4 (16.7-53.6)
ER negative	27 (39%)	WBC (10^9/L)	6.1 (4.8-7.2)
ER expression 0-10%	11 (16%)	RBC (10^9/L)	4.02 (3.84-4.3)
ER expression 10-60%	7 (10%)	HB (g/L)	120 (110-129)
ER expression 60-100%	24 (35%)	Bilirubin, total (umol/L)	10.1 (8.7-12.3)
PR status	35 (51%)	Total protein (g/L)	67.3 (65-70.1)
Her2 status	14 (20%)	ALT(U/L)	15 (11-23)
Limb	29 (42%)	ALP(U/L)	96 (75-124)
Pelvis	9 (13%)	Total bile acid (umol/L)	3.9 (2.7-5.9)
Spine	31 (45%)	AST (U/L)	22 (18-30)
Bone metastasis number≥4	31 (45%)	LDH (U/L)	191 (172-233)
Pathological fracture	32 (46%)	Total cholesterol (mmol/L)	4.6 (4.05-5.25)
Palliative surgery	37 (54%)	Ca (mmol/L)	2.26 (2.19-2.32)
Radical surgery	32 (46%)	Mg (mmol/L)	0.88 (0.82-0.92)
LN metastasis	11 (16%)	Urea nitrogen (umol/L)	4.7 (3.7-5.8)
Brain metastasis	5 (7%)	Creatinine (umol/L)	50 (43-55)
Liver metastasis	3 (4%)	Uric acid (umol/L)	268 (220-334)
Lung metastasis	9 (13%)	Glucose (mmol/L)	4.95 (4.49-5.25)
Other organ metastasis	2 (3%)		
Chemotherapy for metastasis	44 (64%)		
Radiotherapy for metastasis	33 (48%)		
Endocrine therapy for metastasis	24 (35%)		

ER, estrogen receptor; PR, progesterone receptor; LN, lymph node; CEA, carcinoembryonic antigen; CA125, Carbohydrate antigen125; CA153, Carbohydrate antigen153; WBC, white blood cell count; RBC, red blood cell count; HB, hemoglobin; ALT, alanine aminotransferase; ALP, alkaline phosphatase; AST, Aspartate aminotransferase; LDH, lactate dehydrogenase; Ca, calcium; Mg, magnesium.

### Multivariate Cox Regression for Postoperative Mortality

**Figure 3:**
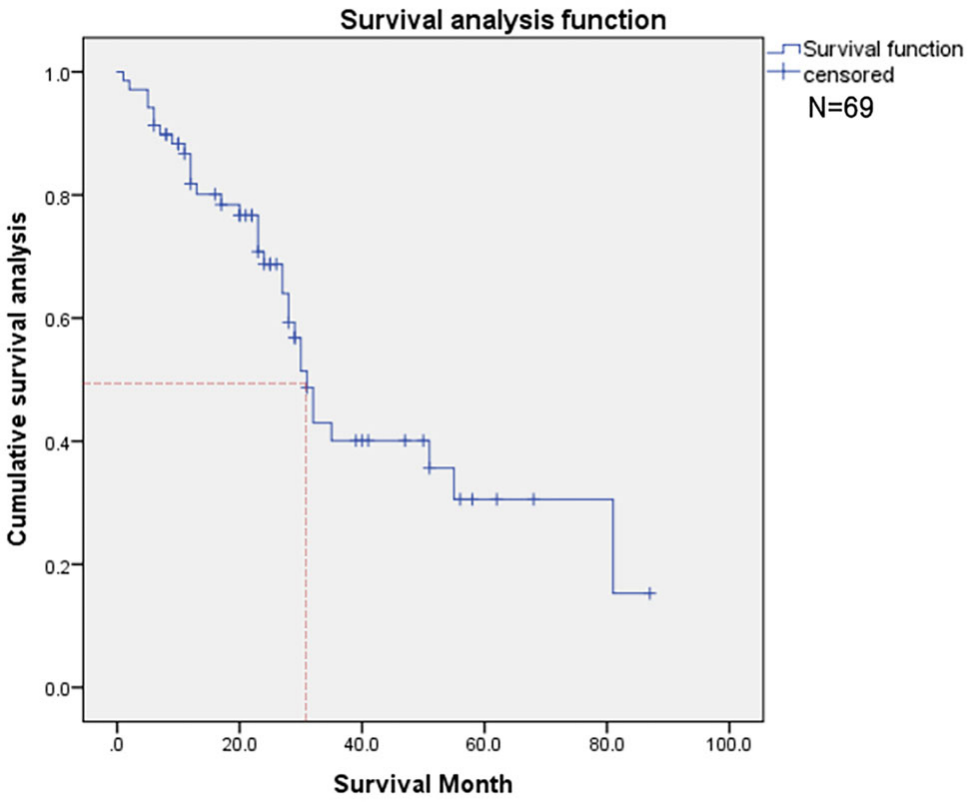
This figure shows the overall survival of patients (N=69).

Thirty-three deaths were found in this time, and the 50% survival rate is around 30 months (**Figure 3**). Among the probable variables (p <0.1 in univariate analysis), we excluded the associated collinearity factor and controlled for relevant confounding variables. We found that radiotherapy for primary cancer (hazard ratio [HR], 3.02; confidence interval [CI], 1.14-8.01, p=0.027), subtype of BC (HR, 2.1; CI,1.4-3.2, p<0.001), multiple bone metastases(HR, 1.55; CI, 0.92-2.60; p=0.098), LN metastasis(HR, 2.80; CI, 1.08-7.22; p=0.034), higher serum ALP(HR, 1.005; CI, 1.001-1.008; p=0.009), CA125(HR, 1.005; CI, 1.001-1.008; p=0.009), LDH(HR, 1.007; CI, 1.002-1.012; p=0.003) were associated with postoperative death (**Table 2**).

**Table 2:** Multivariate Cox regression for postoperative mortality.

Categories	Variables	Univariate analysis	Multivariate analysis	
		p value	HR (95% CI)	p value
Adjuvant therapy for primary cancer	Chemotherapy	0.45		
	Radiotherapy	0.043	3.02 (1.14-8.01)	0.027
	Endocrine therapy	0.9		
Subtype	(Luminal A=1, LuminalB=2, Her2-enhanced=3, Triple-negative=4)	<0.001	2.1 (1.4-3.2)	<0.001
location	Limb	0.88		
	Pelvis	0.44		
	Spine	0.49		
Bone condition	multiple bone metastases (1metastasis=1, 2-3metastases=2, ≥4metastases=3)	<0.001	1.55 (0.92-2.60)	0.098
	Pathological fracture	0.035	–	–
	Surgery methods (Palliative surgery=1, Radical surgery=2)	0.036	–	–
Viscera metastasis	LN metastasis	0.061	2.80 (1.08-7.22)	0.034
	Brain metastasis	0.041	–	–
	Liver metastasis	0.002	–	–
	Lung metastasis	0.012	–	–
	Other organ metastasis	0.036	–	–
Adjuvant therapy for metastasis	Chemotherapy for metastasis	0.12		
	Radiotherapy for metastasis	0.71		
	Endocrine therapy for metastasis	0.98		
Age	Age at primary cancer n ≤ 49 = 0, n>49 = 1	0.18		
	Age at metastasis n ≤ 53 = 0, n>53 = 1	0.31		
	Disease free interval(DFI) n ≤ 5 = 0, n>5 = 1	0.53		
Tumor marker	CEA	0.57		
	CA125	<0.001	1.005 (1.001-1.008)	0.009
	CA153	0.98		
Serum biomarkers	WBC	0.28		
	RBC	0.15		
	Hemoglobin(HB)	0.004	–	–
	Bilirubin, total	0.28		
	Total protein	0.55		
	ALT	0.75		
	ALP	0.002	–	–
	Total bile acid	0.7		
	AST	0.37		
	LDH	<0.001	1.007 (1.002-1.012)	0.003
	Total cholesterol	0.33		
	Ca	0.52		
	P	0.33		
	Urea nitrogen	0.4		
	Creatinine	0.84		
	Uric acid	0.76		
	Glucose	0.85		

HR, hazard ratio; CI, confidence interval; LN, lymph node; CEA, carcinoembryonic antigen; CA125, Carbohydrate antigen125; CA153, Carbohydrate antigen153; WBC, white blood cell count; RBC, red blood cell count; HB, hemoglobin; ALT, alanine aminotransferase; ALP, alkaline phosphatase; AST, Aspartate aminotransferase; LDH, lactate dehydrogenase; Ca, calcium; Mg, magnesium.

**Figure 4:**
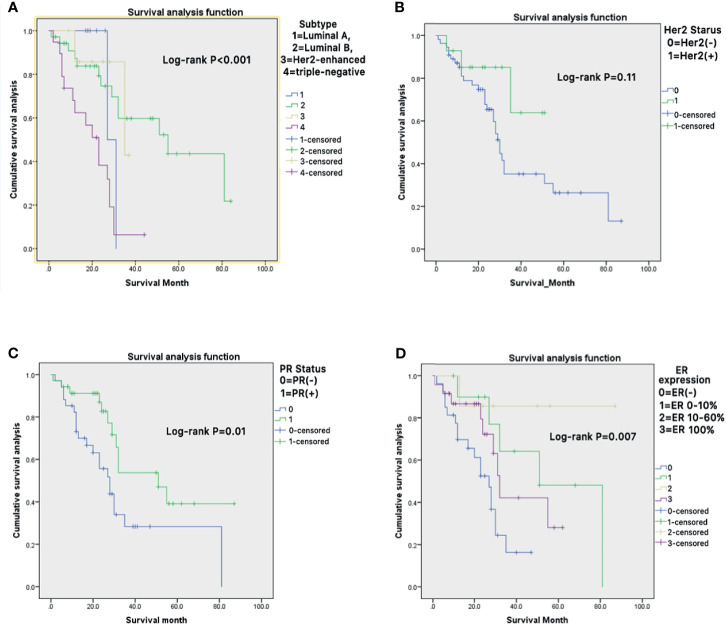
**(A–D)** Kaplan-Meier survival curves were generated to evaluate the different subtypes of metastatic BC **(A)**, receptor molecules **(B, C)**, and their expression **(D)** impacts on the survival prognosis of patients.

Kaplan-Meier curve showed that different subtypes of metastatic BC, receptor molecules, and their expression had a significant impact on the survival prognosis of patients (**Figures 4A–D**).

### 3-Year Survival Risk Prediction Model

**Table 3** summarizes the development of the 3-year survival risk multivariate Cox regression. As shown in the table, each prespecified predictor had a significant (P<0.01) univariable linear relationship with the primary outcome. Considering the small sample size, we performed 100 bootstrapping to establish the risk prediction model.

**Table 3:** 3-year survival risk prediction model.

	Univariable model		Multivariable (final model)		
	HR (95% CI)	p value	HR (95% CI)	p value	
Subtype of metastatic BC	1.8 (1.2-2.7)	0.002	1.65 (1.11-2.46)	0.014	
Multiple bone metastasis	5.1 (1.9-13.3)	0.001	2.94 (1.08-8.01)	0.035	
Pathological fracture	2.1 (1.0-4.3)	0.05	–	–	
Organ metastasis	4.4 (2.1-9.4)	0.0001	3.75 (1.69-8.32)	0.001	
Chemotherapy	0.54 (0.26-1.12)	0.09	–	–	
Age at primary cancer	1 (1-1.1)	0.06	–	–	
Age at metastasis cancer	1 (1.0-1.06)	0.02	–	–	
CEA	1 (0.99-1.01)	0.07	–	–	
CA199	1 (0.99-1.01)	0.08	–	–	
CA125	1 (1.0-1.01)	0.002	–	–	
Hemoglobin(HB)	0.97 (0.94-0.99)	0.005	–	–	
Bilirubin, total	1.1 (1.01-1.15)	0.02	–	–	
ALP	1 (1.0-1.01)	0.005	–	–	Included in the final model
LDH	1 (1.0-1.01)	0.0005	1.005 (1.001-1.009)	0.017	

HR, hazard ratio; CI, confidence interval; CEA, carcinoembryonic antigen; CA125, Carbohydrate antigen125; CA153, Carbohydrate antigen153; WBC, white blood cell count; RBC, red blood cell count; HB, hemoglobin; ALT, alanine aminotransferase; ALP, alkaline phosphatase; LDH, lactate dehydrogenase; Ca, calcium; Mg, magnesium.

**Figure 5:**
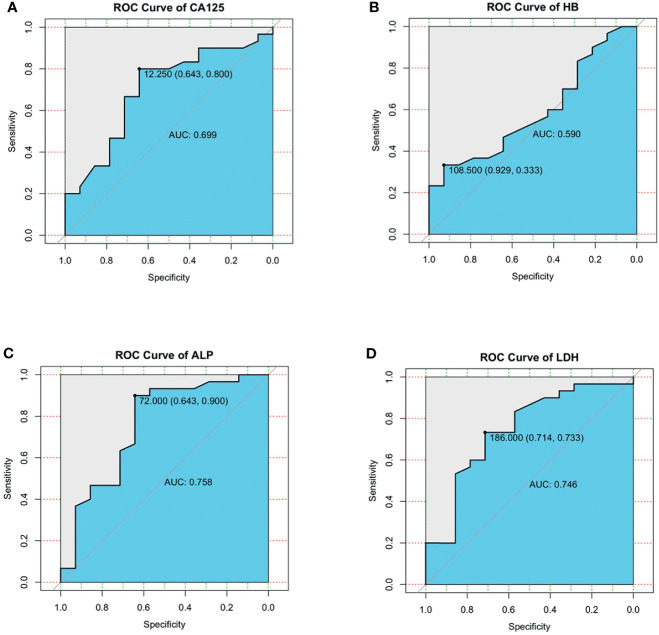
**(A–D)** ROC curves were generated to evaluate the cutoff value of serum CA125 **(A)**, serum HB **(B)**, serum ALP **(C)**, serum LDH **(D)**.

For the possible continuous variables in the single factor, we plot the receiver operating characteristic (ROC) curve. The area under the ROC curve (AUC) of serum CA125 for 3-year survival was 0.699(95% CI, 0.52-0.87). At the cutoff value of 12.25, sensitivity was 80.0%, and specificity was 64.3% (**Figure 5A**). AUC of serum HB for 3-year survival was 0.590(95% CI, 0.41-0.77). At the cutoff value of 108.5, sensitivity was 33.3%, and specificity was 92.9% (**Figure 5B**). AUC of serum ALP for 3-year survival was 0.758(95% CI, 0.58-0.93). At the cutoff value of 72.0, sensitivity was 90.0%, and specificity was 64.3% (**Figure 5C**). AUC of serum LDH for 3-year survival was 0.746(95% CI, 0.58-0.91). At the cutoff value of 186.0, sensitivity was 73.3%, and specificity was 71.4% (**Figure 5D**).

**Figure 6:**
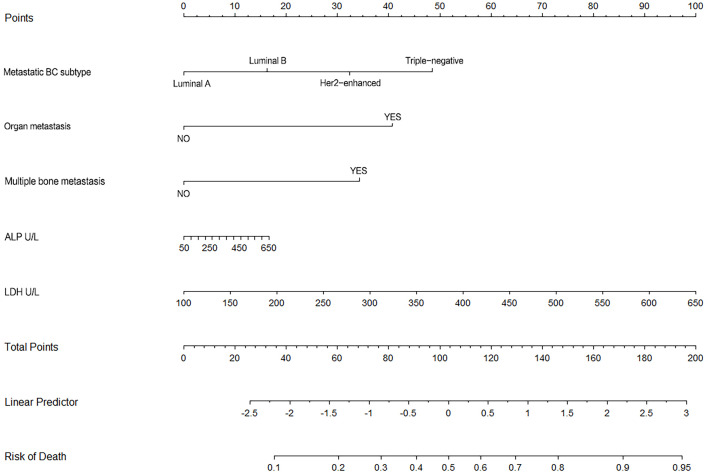
This figure shows the nomogram of for 3-year survival for the patients undergoing BC surgery and bone metastasis surgery.

**Figure 7:**
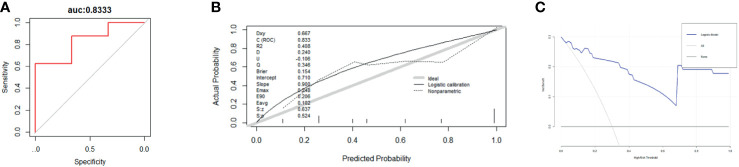
**(A–C)** These figures show the ROC curves **(A)**, calibration plot **(B)**, and decision curve analysis **(C)** for 3-year survival for the patients undergoing BC surgery and bone metastasis surgery. (N = 44).

Nomogram (**Figure 6**) based on these independent variables and serum ALP level was established to maximize predictive power. The optimism-corrected C-statistic (**Figure 7A**) of the predictive model was 0.83(95% CI 0.56-1). **Figure 7B** presents a graphical representation of calibration. We subsequently demonstrate our model using decision curve analysis (**Figure 7C**).

**Figure 8:**
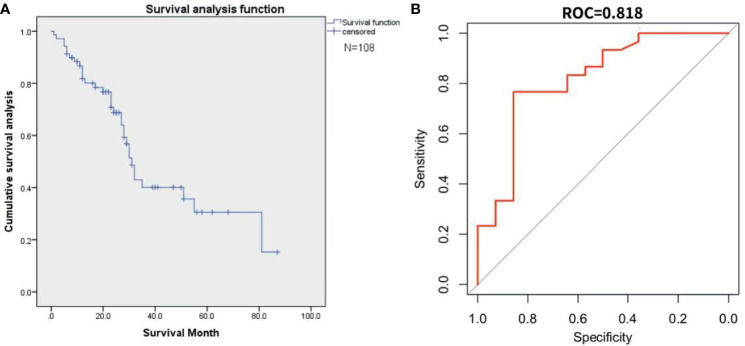
This figure represents the external validation result. **(A)** The survival curve from the Surveillance, Epidemiology, and End Results(SEER) database. **(B)** The AUC curve for external validation of SEER database.

**Figure 8** represents the result of external validation. We drew the survival curve (**Figure 8A**) and used the all input method to verify the fitting of the two databases (**Figure 8B**). the AUC of external validation was 0.82 (95%CI, 0.65-0.90).

## Discussion

### Background and Rationale

The occurrence of metastatic bone disease indicates that cancer has reached its final stage (3, 4). Surgical treatment of metastatic disease aims to alleviate pain and restore biomechanical stability. Besides, how to predict the prognosis of this group of people accurately is rarely known by people. To contribute some valuable insights into this challenging disease’s prognosis and treatment strategies, we made a retrospective analysis about breast cancer patients with postoperative bone metastases from our institution.

### Risk Factors for Postoperative Mortality

We classified several independent clinical factors associated with postoperative mortality in patients with bone metastasis, including the radiotherapy status of primary loci, the subtype of metastatic BC, multiple bone metastases, presence of LN metastasis, serum CA125 and LDH level.

Firstly, the primary site decides the prognosis of metastatic bone disease. The subtype of the BC is the most significant risk factor in this study (7).Hormone receptor status and her2 status have become significant factors in the classification of breast cancer patients to assess prognosis and determine the appropriate treatment (23). Studies pointed that Triple-negative breast cancer leads to decreased worse overall survival than non-triple-negative breast cancer. Although all breast cancer subtypes are susceptible to bone metastasis, the luminal type is the most common (24). Clinically, the biomarkers of the primary tumor guide the treatment of patients with metastatic breast cancer. Some studies had shown that there might be clinically significant discordance of ER, PR, and her2 status when comparing primary breast tumors with metastasis, which might impact overall survival (18, 25, 26). Therefore, patients’ management and survival may be improved by a biopsy of the metastatic lesions before surgery potentially (25).

In Addition, the presence of extraosseous disease and the extent and tempo of the bone disease are potent predictors of prognosis (7). The presence of multiple bone metastases is associated with a poor prognosis (27). The survival was longer for single bone metastasis compared to multiple bone metastases (28, 29). In patients with cancer, fractures often indicate a worsening survival prognosis (30). We found that patients with pathological fractures had a worse prognosis than those without fractures. Since these patients often suffer from pain and limited movement, pathological fractures indicate poor quality of life. Whenever possible, a multidisciplinary discussion should guide treatment decisions for metastatic bone fractures to ensure that the combination of surgery and medication is optimal (31–33).

It was broadly believed that receiving radiotherapy might reduce pain and obtain reasonable local control. Several researches have reported that breast radiation therapy improves survival in patients with metastasis (34, 35). Our study unveiled that BC patients with bone metastasis who received radiotherapy could achieve survival benefits in the same way. As for metastatic BC, chemotherapy was usually applied because it decreased cancer-related complications, improved life quality, and prolonged overall survival (36). However, chemotherapy was not identified as an important predictor in our multivariate analysis, which might be related to the specific chemotherapy regimen. Due to the limitations of difficult follow-up and incomplete information, we did not conduct further studies. Endocrine therapy is the main method of early treatment for breast cancer for hormone receptor positive patients (37). Our findings suggested that this might not be an independent prognostic factor. Some studies showed that the relationship between endocrine therapy for breast cancer and survival outcomes and DFI remained to be further investigated (38, 39). However, endocrine treatment for breast cancer had also been shown to affect DFI rather than survival (40, 41), consistent with our findings (**Supplementary Material**).

We cannot manage for this analysis, it is not clear if age is a risk factor or linked to other diseases (42). However, we unveiled an increased risk of mortality after operation for older patients that the age at diagnosis of BC was related to poor prognosis in the univariate analysis. Rüdiger J et al. (43) found that the age of 60 as the dividing line was statistically significant. The criteria for age grouping may vary among study groups, but most revealed that the advanced age at breast cancer diagnosis is an adverse prognostic factor. The defined age remains unclear. Also, age at diagnosis is a prognostic factor for metastatic breast cancer patients (44). Shorter DFI was associated with worse overall survival and shorter time to progression (45).

Among the current studies, the simple Cox analysis revealed that the prognosis of patients with organ involvement metastases is often worse than that of patients with solely skeletal metastases. However, in the adjusted multiple analysis, this covariate lost its significance in the adjusted multiple analysis. The appearance of additional visceral metastases as a negative predictor for survival was documented in other statements (46, 47). Consistent with these studies, patients with bone-only metastasis had better overall survival than those with visceral metastasis (48, 49).

Several laboratory test values before surgery were considerably associated with patients’ OS. When patients are admitted for surgery, series of preoperative examination will be performed. In this study, low serum HB, elevated serum LDH, CA125, ALP levels were adverse prognostic factors for OS. However, the mechanism of the occurrence of SREs and laboratory test values has not been proven. Elevated LDH level is an indicator of tumor burden in patients with breast cancer. Elevated serum LDH is a poor prognostic biomarker in cancer metabolism. It is a crucial enzyme involved in cancer metabolism, allowing tumor cells to inhibit and altering the tumor microenvironment to evade the immune system simultaneously.  (50) HB was not only a valuable marker for tumor aggressiveness but also played an essential role in distant metastasis, which had been suggested in previous studies (51, 52). In recent studies, we recognized Hb concentration as an independent risk factor associated with bone metastases. Serum total alkaline phosphatase (ALP) exists in human tissues, reflecting osteoblastic activity. It was more evident in patients with aggressive bone metastases (53). Clinically, there is often a significant difference between normal people and patients with metastatic bone cancer. It had been previously confirmed the value of especially bone-derived ALP, a tumor formation marker in patients with bone metastasis. Chen et al. believed that serum ALP > 100.5 U/L concentration was recognized as a risk factor for bone metastasis in breast cancer patients (53). Our study revealed that serum ALP as a hazard factor for bone metastasis in patients with BC was consistent with previous studies. CA125 is a tumor marker for ovarian cancer. However, it rarely evaluated the prognosis of bone metastases in breast cancer (54). In our study, CA125 concentrations were prominently higher in patients with bone metastases who died early after surgery than in patients who survived. Berruti et al. (55) found that patients with CA125 < 35 U/mL had a better prognosis, consisting of our research.

### 3-Year Survival Prediction Model

We selected patients who survived for more than three years or died within three years to construct the model (n=44). We established the nomogram based on these independent variables and serum ALP level to maximize predictive power. Our nomograms can be referred by surgeons to promote the appropriate strategy for those populations. Wang et al. (2) developed a clinical nomogram for probability prediction of female patients with breast cancer and bone metastasis based on seven independent predictors. Marital status, race, age, T stage, tumor grade, ER, PR, HER2, brain metastasis, liver metastasis, lung metastasis, and breast surgery were independent factors for BC bone metastasis, which were revealed by Hou et al.They also constructed a nomogram highly consistent with our results (14). Nomograms had been extensively constructed to predict outcomes for different BC populations and show a significant advantage in their clinical management and strategy (56, 57). The optimism-corrected C-statistic of the predictive model was 0.83(95% CI 0.56-1). The calibration plot shows excellent overall agreement between the predicted and observed 3-year risk, which presented an apparent correlation between prediction and actual observation.

To assess the implications of our model in clinical practice, we subsequently demonstrate our model using decision curve analysis. The effect of our model is quite excellent. Within an extensive PT range, the benefits are higher than those of the extreme curve, so the optional PT range is relatively extensive and reliable.

### External Validation

In the study of the SEER database, we found that only 108 cases had matched the information of our enrolled patients. The common reference indexes were organ metastasis, hormone receptor, age, radiotherapy and chemotherapy of primary foci, etc., which indicated that the database parameters used for verification were relatively limited, not as detailed as our database. When the external validation was modeled with relevant independent factors, overfitting also occurred. Therefore, we adopted the all-input (the common parameter) method to verify the reliability of the data. The ROC was 0.82, which reflected a reliable external validation result. Interestingly, after plotting the survival curves using the SEER database, we discovered that the overall survival trend in SEER database matched the trend in our institutional data.

### Limitation

This study had some unavoidable limitations. This is a single-centre retrospective study with a limited sample size, so we need a lot of prospective external data validation. The included indicators of our Database research and the public Database (SEER Database) were also limited. For instance, laboratory test parameters were unavailable in the SEER Database. Although external validation initially showed a good fit between the two databases, further prospective clinical trials are still of importance. Second, only a potential association between the variables and prognosis was displayed in our study. However, the mechanism could not be explained because the goal of our study was to look for indicators that could predict patient survival. Although many variables were estimated, there might be variables not incorporated, such as the ECOG score table and the specific chemotherapy regimens. Many patients underwent breast cancer surgery in other hospitals years ago. The detailed information of therapeutic strategy involving chemotherapy regimens was not fully provided. As for the 3-year prediction model, population selection may overestimate mortality, resulting in bias. Additionally, it is unclear that the choice of surgical method is an independent factor in determining patient outcomes. We were inadequate to decide on any previous surgical standards due to the retrospective nature of the study, which may have contributed to selection bias. For example, some patients choose palliative surgery because their overall health is poor. Furthermore, our study involved patients who had undergone surgery for breast cancer and surgery for bone metastases. These prognostic factors may not be fully applicable for patients with untreated bone metastases.

## Conclusions

In conclusion, we found radiotherapy for primary cancer, metastatic tumor type, organ metastasis, multiple bone metastasis, preoperative serum levels of CA125, LDH associated with postoperative death. We were also able to predict the risk of 3-year survival after surgery for breast cancer bone metastasis by tumor type of metastatic site, organ metastasis, multiple bone metastasis, and serum LDH and ALP before surgery. Our study suggests that several clinical features and serological markers can predict the overall survival among the patients who are about to receive bone metastasis surgery after BC surgery. The model can guide the preoperative evaluation and clinical decision-making for patients. What’s more, bone tumor doctors can focus more on these indicators rather than the characteristics of the tempo of the bone disease, which can preliminary judge the prognosis of breast cancer bone metastases. In spite of the hazard factors for early death that need to be confirmed by prospective researchers, non-operative therapy or close monitoring after surgery should be considered for high-risk preoperative patients.

## Data Availability Statement

The original contributions presented in the study are included in the article/**Supplementary Material**. Further inquiries can be directed to the corresponding author.

## Ethics Statement

The studies involving human participants were reviewed and approved by Ethics Committee of the Second Affiliated Hospital of Zhejiang University School of Medicine. Written informed consent for participation was not required for this study in accordance with the national legislation and the institutional requirements.

## Author Contributions

Proposed research topic: ZW. Design and research scheme: GL. Implement the research process: HM. Collect and organize data: HM, FW, YX, and XC. Research and collate literature: WZ and HZ. Design the paper framework: ZNW and TC. Drafted paper: HM and EY. Revised paper: BL. Final Review Paper: ZY. Statistical analysis of: GL. Technical or material support: GL. Instructive support: PL, WT, and HQ. All authors contributed to the article and approved the submitted version.

## Conflict of Interest

The authors declare that the research was conducted in the absence of any commercial or financial relationships that could be construed as a potential conflict of interest.

## Publisher’s Note

All claims expressed in this article are solely those of the authors and do not necessarily represent those of their affiliated organizations, or those of the publisher, the editors and the reviewers. Any product that may be evaluated in this article, or claim that may be made by its manufacturer, is not guaranteed or endorsed by the publisher.

## References

[1] Pore SK Hahm ER Latoche JD Anderson CJ Shuai Y Singh SV. Prevention of Breast Cancer-Induced Osteolytic Bone Resorption by Benzyl Isothiocyanate. Carcinogenesis (2018) 39(2):134–45. doi: 10.1093/carcin/bgx114 PMC5862255 29040431

[2] Wang Z Cheng Y Chen S Shao H Chen X Wang Z. Novel Prognostic Nomograms for Female Patients With Breast Cancer and Bone Metastasis at Presentation. Ann Trans Med (2020) 8(5):197. doi: 10.21037/atm.2020.01.37 PMC7154431 32309344

[3] Fang J Xu Q. Differences of Osteoblastic Bone Metastases and Osteolytic Bone Metastases in Clinical Features and Molecular Characteristics. Clin Trans Oncol (2015) 17(3):173–9. doi: 10.1007/s12094-014-1247-x 25351174

[4] Coleman RE. Metastatic Bone Disease: Clinical Features, Pathophysiology and Treatment Strategies. Cancer Treat Rev (2001) 27(3):165–76. doi: 10.1053/ctrv.2000.0210 11417967

[5] Kuchuk I Hutton B Moretto P Ng T Addison CL Clemons M. Incidence, Consequences and Treatment of Bone Metastases in Breast Cancer Patients—Experience From a Single Cancer Centre. J Bone Oncol (2013) 2(4):137–44. doi: 10.1016/j.jbo.2013.09.001 PMC4723382 26909284

[6] Svensson E Christiansen CF Ulrichsen SP Rorth MR Sorensen H. Survival After Bone Metastasis by Primary Cancer Type: A Danish Population-Based Cohort Study. BMJ Open (2017) 7(9):e016022. doi: 10.1136/bmjopen-2017-016022 PMC5595184 28893744

[7] Coleman RE. Clinical Features of Metastatic Bone Disease and Risk of Skeletal Morbidity. Clin Cancer Res (2006) 12(20 Pt 2):6243s–9s. doi: 10.1158/1078-0432.CCR-06-0931 17062708

[8] Kim JH Seo SW Chung CH. What Factors Are Associated With Early Mortality in Patients Undergoing Femur Surgery for Metastatic Lung Cancer? Clin Orthopaedics Related Res (2018) 476(9):1815–22. doi: 10.1007/s11999.0000000000000101 PMC6259786 30794217

[9] Tan Y Li X Chen H Hu Y Jiang M Fu J. Hormone Receptor Status may Impact the Survival Benefit of Surgery in Stage Iv Breast Cancer: A Population-Based Study. Oncotarget (2016) 7(43):70991–1000. doi: 10.18632/oncotarget.11235 PMC5342604 27542240

[10] Akay CL Ueno NT Chisholm GB Hortobagyi GN Woodward WA Alvarez RH. Primary Tumor Resection as a Component of Multimodality Treatment may Improve Local Control and Survival in Patients With Stage IV Inflammatory Breast Cancer. Cancer (2014) 120(9):1319–28. doi: 10.1002/cncr.28550 PMC4278570 24510381

[11] Hou N Yi J Wang Z Yang L Wu Y Huang M. Development and Validation of a Risk Stratification Nomogram for Predicting Prognosis in Bone Metastatic Breast Cancer: A Population-Based Study. Med (Baltimore) (2021) 100(6):e24751. 10.1097/MD.0000000000024751 PMC10545337 33578627

[12] Tu Q Hu C Zhang H Peng C Kong M Song M. Establishment and Validation of Novel Clinical Prognosis Nomograms for Luminal A Breast Cancer Patients With Bone Metastasis. BioMed Res Int (2020) 2020:1972064. doi: 10.1155/2020/1972064 33490234 PMC7787749

[13] Liu D Wu J Lin C Andriani L Ding S Shen K. Breast Subtypes and Prognosis of Breast Cancer Patients With Initial Bone Metastasis: A Population-Based Study. Front Oncol (2020) 10:580112. doi: 10.3389/fonc.2020.580112 33344236 PMC7739957

[14] Xiong Y Shi X Hu Q Wu X Long E Bian Y. A Nomogram for Predicting Survival in Patients With Breast Cancer Liver Metastasis: A Population-Based Study. Front Oncol (2021) 11:600768. doi: 10.3389/fonc.2021.600768 34150607 PMC8206538

[15] Mirels H. Metastatic Disease in Long Bones: A Proposed Scoring System for Diagnosing Impending Pathologic Fractures. 1989. Clin Orthop Relat Res (2003) 415(Suppl):S4–13. doi: 10.1097/01.blo.0000093045.56370.dd 14600587

[16] Tomita K Kobayashi T Yoshida A Murakami H Akamaru T. Surgical Strategy for Spinal Metastases. Spine (Phila Pa 1976) (2001) 26(3):298–306. doi: 10.1097/00007632-200102010-00016 11224867

[17] Perou CM Sorlie T Eisen MB van de Rijn M Jeffrey SS Rees CA. Molecular Portraits of Human Breast Tumours. Nature (2000) 406(6797):747–52. doi: 10.1038/35021093 10963602

[18] Yao Z Lu LJ Wang RJ Jin LB Liu SC Li HY Ren GS. Discordance and Clinical Significance of ER, PR, and HER2 Status Between Primary Breast Cancer and Synchronous Axillary Lymph Node Metastasis. Med Oncol (2014) 31(1). doi: 10.1007/s12032-013-0798-y 24307349

[19] Moons KG Altman DG Reitsma JB Ioannidis JP Macaskill P Steyerberg E. Transparent Reporting of a Multivariable Prediction Model for Individual Prognosis or Diagnosis (TRIPOD): Explanation and Elaboration. Ann Intern Med (2015) 162(1):W1–73. doi: 10.7326/M14-0698 25560730

[20] Bongers MER Karhade AV Setola E Gambarotti M Groot OQ Erdogan KE. How Does the Skeletal Oncology Research Group Algorithm’s Prediction of 5-Year Survival in Patients With Chondrosarcoma Perform on International Validation? Clin Orthopaedics Related Res (2020) 478(10):2300–8. doi: 10.1097/CORR.0000000000001305 PMC7491905 32433107

[21] Steyerberg EW Vergouwe Y. Towards Better Clinical Prediction Models: Seven Steps for Development and an ABCD for Validation. Eur Heart J (2014) 35(29):1925–31. doi: 10.1093/eurheartj/ehu207 PMC4155437 24898551

[22] Van Calster B Vickers AJ. Calibration of Risk Prediction Models: Impact on Decision-Analytic Performance. Med Decis Making (2015) 35(2):162–9. doi: 10.1177/0272989X14547233 25155798

[23] Schnitt SJ. Classification and Prognosis of Invasive Breast Cancer: From Morphology to Molecular Taxonomy. Mod Pathol (2010) 23(Suppl 2):S60–4. doi: 10.1038/modpathol.2010.33 20436504

[24] Li X Yang J Peng L Sahin AA Huo L Ward KC. Triple-Negative Breast Cancer has Worse Overall Survival and Cause-Specific Survival Than Non-Triple-Negative Breast Cancer. Breast Cancer Res Treat (2017) 161(2):279–87. doi: 10.1007/s10549-016-4059-6 27888421

[25] Lindstrom LS Karlsson E Wilking UM Johansson U Hartman J Lidbrink EK. Clinically Used Breast Cancer Markers Such as Estrogen Receptor, Progesterone Receptor, and Human Epidermal Growth Factor Receptor 2 Are Unstable Throughout Tumor Progression. J Clin Oncol (2012) 30(21):2601–8. doi: 10.1200/JCO.2011.37.2482 22711854

[26] Dieci MV Barbieri E Piacentini F Ficarra G Bettelli S Dominici M. Discordance in Receptor Status Between Primary and Recurrent Breast Cancer has a Prognostic Impact: A Single-Institution Analysis. Ann Oncol (2013) 24(1):101–8. doi: 10.1093/annonc/mds248 23002281

[27] Bindels BJJ Thio QCBS Raskin KA Ferrone ML Lozano Calderon SA Schwab JH. Thirty-Day Postoperative Complications After Surgery For Metastatic Long Bone Disease Are Associated With Higher Mortality at 1 Year. Clin Orthopaedics Related Res (2020) 478(2):306–18. doi: 10.1097/CORR.0000000000001036 PMC7438145 31714410

[28] Zhang L Gong Z. Clinical Characteristics and Prognostic Factors in Bone Metastases From Lung Cancer. Med Sci Monit (2017) 23:4087–94. doi: 10.12659/MSM.902971 PMC5580519 28835603

[29] Janssen SJ Kortlever JT Ready JE Raskin KA Ferrone ML Hornicek FJ. Complications After Surgical Management of Proximal Femoral Metastasis: A Retrospective Study of 417 Patients. J Am Acad Orthop Surg (2016) 24(7):483–94. doi: 10.5435/JAAOS-D-16-00043 27227983

[30] Anract P Biau D Boudou-Rouquette P. Metastatic Fractures of Long Limb Bones. Orthopaedics Traumatol: Surg Res (2017) 103(1):S41–51. doi: 10.1016/j.otsr.2016.11.001 28089230

[31] Eastley N Newey M Ashford RU. Skeletal Metastases - the Role of the Orthopaedic and Spinal Surgeon. Surg Oncol (2012) 21(3):216–22. doi: 10.1016/j.suronc.2012.04.001 22554913

[32] Capanna R Piccioli A Di Martino A Daolio PA Ippolito V Maccauro G. Management of Long Bone Metastases: Recommendations From the Italian Orthopaedic Society Bone Metastasis Study Group. Expert Rev Anticancer Ther (2014) 14(10):1127–34. doi: 10.1586/14737140.2014.947691 25151850

[33] Janssen SJ van Dijke M Ready JE Raskin KA Ferrone ML Hornicek FJ. 2015 Marshall Urist Young Investigator Award: Prognostication in Patients With Long Bone Metastases: Does a Boosting Algorithm Improve Survival Estimates? Clin Orthop Relat Res (2015) 473(10):3112–21. doi: 10.1007/s11999-015-4446-z PMC4562931 26155769

[34] Le Scodan R Kim JW Choi J Sohn J Kim SI Park S. Breast Cancer With Synchronous Metastases: Survival Impact of Exclusive Locoregional Radiotherapy. J Clin Oncol (2009) 27(9):1375–81. doi: 10.1200/JCO.2008.19.5396 19204198

[35] Choi SH Kim JW Choi J Sohn J Kim SI Park S. Locoregional Treatment of the Primary Tumor in Patients With De Novo Stage IV Breast Cancer: A Radiation Oncologist's Perspective. Clin Breast Cancer (2018) 18(2):e167–78. doi: 10.1016/j.clbc.2017.06.002 28689012

[36] Mehta RS Barlow WE Albain KS Vandenberg TA Dakhil SR Tirumali N. Overall Survival With Fulvestrant Plus Anastrozole in Metastatic Breast Cancer. N Engl J Med (2019) 380(13):1226–34. doi: 10.1056/NEJMoa1811714 PMC6885383 30917258

[37] D Souza A Spicer D Lu J. Overcoming Endocrine Resistance in Metastatic Hormone Receptor-Positive Breast Cancer. J Hematol Oncol (2018) 11(1). doi: 10.1186/s13045-018-0620-6 PMC5996460 29891002

[38] Harbeck N Gnant M. Breast Cancer. Lancet (2017) 389(10074):1134–50. doi: 10.1016/S0140-6736(16)31891-8 27865536

[39] Reinbolt RE Mangini N Hill JL Levine LB Dempsey JL Singaravelu J. Endocrine Therapy in Breast Cancer: The Neoadjuvant, Adjuvant, and Metastatic Approach. Semin Oncol Nurs (2015) 31(2):146–55. doi: 10.1016/j.soncn.2015.02.002 25951743

[40] Maughan KL Lutterbie MA Ham PS. Treatment of Breast Cancer. Am Fam Physician (2010) 81(11):1339–46. 20521754

[41] Early Breast Cancer Trialists' Collaborative Group (EBCTCG). Effects of Chemotherapy and Hormonal Therapy for Early Breast Cancer on Recurrence and 15-Year Survival: An Overview of the Randomised Trials. Lancet (2005) 365(9472):1687–717. doi: 10.1016/S0140-6736(05)66544-0 15894097

[42] Nathan SS Healey JH Mellano D Hoang B Lewis I Morris CD. Survival in Patients Operated on for Pathologic Fracture: Implications for End-Of-Life Orthopedic Care. J Clin Oncol (2005) 23(25):6072–82. doi: 10.1200/JCO.2005.08.104 16135474

[43] Weiss RJ Tullberg E Forsberg JA Bauer HC Wedin R. Skeletal Metastases in 301 Breast Cancer Patients: Patient Survival and Complications After Surgery. Breast (2014) 23(3):286–90. doi: 10.1016/j.breast.2014.02.012 24684891

[44] Chen MT Sun HF Zhao Y Fu WY Yang LP Gao SP. Comparison of Patterns and Prognosis Among Distant Metastatic Breast Cancer Patients by Age Groups: A SEER Population-Based Analysis. Sci Rep (2017) 7(1):9254. doi: 10.1038/s41598-017-10166-8 28835702 PMC5569011

[45] Abdel-Rahman O. Outcomes of Metastatic Breast Cancer Patients in Relationship to Disease-Free Interval Following Primary Treatment of Localized Disease; a Pooled Analysis of Two Clinical Trials. Breast J (2019) 25(5):823–8. doi: 10.1111/tbj.13346 31134726

[46] Turner NC Finn RS Martin M Im SA DeMichele A Ettl J. Clinical Considerations of the Role of Palbociclib in the Management of Advanced Breast Cancer Patients With and Without Visceral Metastases. Ann Oncol (2018) 29(3):669–80. doi: 10.1093/annonc/mdx797 PMC5888946 29342248

[47] Kast K Link T Friedrich K Petzold A Niedostatek A Schoffer O. Impact of Breast Cancer Subtypes and Patterns of Metastasis on Outcome. Breast Cancer Res Treat (2015) 150(3):621–9. doi: 10.1007/s10549-015-3341-3 25783184

[48] Gong Y Zhang J Ji P Ling H Hu X Shao ZM. Incidence Proportions and Prognosis of Breast Cancer Patients With Bone Metastases at Initial Diagnosis. Cancer Med (2018) 7(8):4156–69. doi: 10.1002/cam4.1668 PMC6089179 29984914

[49] Jacobson AF Shapiro CL Van den Abbeele AD Kaplan WD. Prognostic Significance of the Number of Bone Scan Abnormalities at the Time of Initial Bone Metastatic Recurrence in Breast Carcinoma. Cancer (2001) 91(1):17–24. doi: 10.1002/1097-0142(20010101)91:1<17::AID-CNCR3>3.0.CO;2-K 11148555

[50] Ding J Karp JE Emadi A. Elevated Lactate Dehydrogenase (LDH) Can Be a Marker of Immune Suppression in Cancer: Interplay Between Hematologic and Solid Neoplastic Clones and Their Microenvironments. Cancer Biomarkers (2017) 19(4):353–63. doi: 10.3233/CBM-160336 PMC13020749 28582845

[51] Kume H Kakutani S Yamada Y Shinohara M Tominaga T Suzuki M. Prognostic Factors for Renal Cell Carcinoma With Bone Metastasis: Who Are the Long-Term Survivors? J Urol (2011) 185(5):1611–4. doi: 10.1016/j.juro.2010.12.037 21419440

[52] Huang P Lan M Peng AF Yu QF Chen WZ Liu ZL. Serum Calcium, Alkaline Phosphotase and Hemoglobin as Risk Factors for Bone Metastases in Bladder Cancer. PloS One (2017) 12(9):e0183835. doi: 10.1371/journal.pone.0183835 28902911 PMC5597169

[53] Chen WZ Shen JF Zhou Y Chen XY Liu JM Liu ZL. Clinical Characteristics and Risk Factors for Developing Bone Metastases in Patients With Breast Cancer. Sci Rep (2017) 7(1):11325. doi: 10.1038/s41598-017-11700-4 28900285 PMC5595860

[54] Meyer T Rustin GJ. Role of Tumour Markers in Monitoring Epithelial Ovarian Cancer. Br J Cancer (2000) 82(9):1535–8. doi: 10.1054/bjoc.2000.1174 PMC2363391 10789720

[55] Berruti A Tampellini M Torta M Buniva T Gorzegno G Dogliotti L. Prognostic Value in Predicting Overall Survival of Two Mucinous Markers: CA 15-3 and CA 125 in Breast Cancer Patients at First Relapse of Disease. Eur J Cancer (1994) 30A(14):2082–4. doi: 10.1016/0959-8049(94)00356-A 7857707

[56] Sun W Cheng M Zhou H Huang W Qiu Z. Nomogram Predicting Cause-Specific Mortality in Nonmetastatic Male Breast Cancer: A Competing Risk Analysis. J Cancer (2019) 10(3):583–93. doi: 10.7150/jca.28991 PMC6360428 30719155

[57] Zhao S Shen W Du R Luo X Yu J Zhou W. Three Inflammation-Related Genes Could Predict Risk in Prognosis and Metastasis of Patients With Breast Cancer. Cancer Med (2019) 8(2):593–605. doi: 10.1002/cam4.1962 30632703 PMC6382731

