# Supplementary material for: Clinical Features and Serological Markers Risk Model Predicts Overall Survival in Patients Undergoing Breast Cancer and Bone Metastasis Surgeries
Source: Front Oncol. 2021 Sep 17;11:693689. doi: 10.3389/fonc.2021.693689 (PMC8484887; doi:10.3389/fonc.2021.693689)
Supplement: Supplementary file 1 [file Table_1.docx]

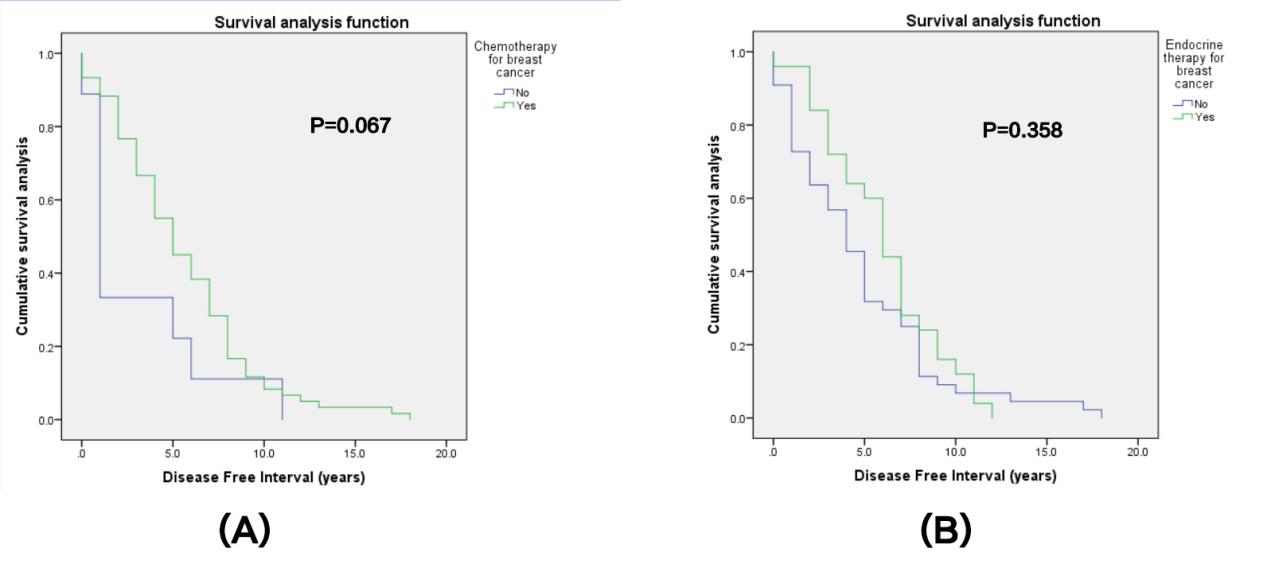


1. represents the relationship between chemotherapy and disease free interval (DFI).
2. represents the relationship between endocrine therapy and DFI.


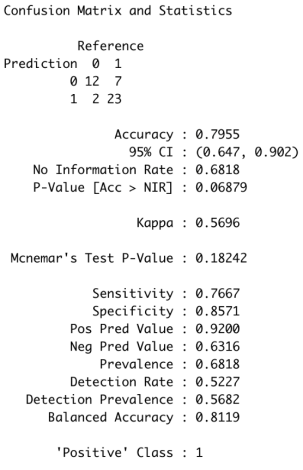
 (C)

This figure represents the process of external validation.
